# Application of Blood Flow Restriction to Optimize Exercise Countermeasures for Human Space Flight

**DOI:** 10.3389/fphys.2019.00033

**Published:** 2019-01-25

**Authors:** Michael Behringer, Christina Willberg

**Affiliations:** Institute of Sports Sciences, Goethe University Frankfurt, Frankfurt, Germany

**Keywords:** human space flight, exercise countermeasure, adaptations to microgravity, BFR training, space adaptations

## Abstract

In recent years there has been a strong increase in publications on blood flow restriction (BFR) training. In particular, the fact that this type of training requires only low resistance to induce muscle strength and mass gains, makes BFR training interesting for athletes and scientists alike. For the same reason this type of training is particularly interesting for astronauts working out in space. Lower resistance during training would have the advantage of reducing the risk of strain-induced injuries. Furthermore, strength training with lower resistances would have implications for the equipment required for training under microgravity conditions, as significantly lower resistances have to be provided by the training machines. Even though we are only about to understand the effects of blood flow restriction on exercise types other than low-intensity strength training, the available data indicate that BFR of leg muscles is also able to improve the training effects of walking or running at slow speeds. The underlying mechanisms of BFR-induced functional and structural adaptations are still unclear. An essential aspect seems to be the premature fatigue of Type-I muscle fibers, which requires premature recruitment of Type-II muscle fibers to maintain a given force output. Other theories assume that cell swelling, anabolic hormones, myokines and reactive oxygen species are involved in the mediation of BFR training-related effects. This review article is intended to summarize the main advantages and disadvantages, but also the potential risks of such training for astronauts.

## Introduction

Human Spaceflight is still a technical and life science challenge. It is well known, that there are several hazards for the human body in space due to microgravity exposure, radiation, sensory deprivation, the disruption of circadian rhythms as well as the artificial environment (Dayanandan, 2011). In short- and long-term space flights, microgravity has physiological effects on the cardiovascular and musculoskeletal system, solving in compromised aerobic capacity, a decrease of bone density and mineral content as well as muscular atrophy and loss of muscular strength (Widrick et al., 1999; Fitts et al., 2001; Sibonga et al., 2007; Trappe et al., 2009). In order to counteract these changes, special training equipment for use in microgravity was developed and used quite early in the history of manned spaceflight. During the 28- to 84-day Skylab missions in the 1970s, a cycle ergometer was shipped to the international space station (ISS). However, even with a daily training program on the ergometer, the astronauts’ maximum oxygen uptake, muscle mass and bone density was decreased on their return from the Skylab missions (Sawin et al., 1976; Vernikos and Schneider, 2010). More recent data indicate that tendon fibers are also adversely affected by prolonged exposure to microgravity (Galloway et al., 2013; McCrum et al., 2018).

Much time has passed since these early Skylab missions and the training equipment for use in microgravity has been continuously refined since then (for review see Loerch, 2015). In the belief that high mechanical forces are required as training stimuli to prevent the musculoskeletal system from being de-adapted, a special focus has been placed on the development of exercise devices that can realize these high mechanical forces (Loerch, 2015). As a result of these efforts, astronauts currently have a multifunctional strength training device the Advanced Resistive Exercise Device (ARED) available, which can realize high resistances of up to up to 110 kg for cable and 270 kg for bar exercises (Trappe et al., 2009). Since it is well documented that resistance training with moderate to high loads is effective in inducing muscle mass and strength gains both on earth (Garber et al., 2011) and under microgravity conditions (Loehr et al., 2011), the availability of the ARED on the ISS is a major step forward for the maintenance of astronauts’ health.

Although, it seems obvious that unloading is best compensated by the application of training stimuli that primarily place mechanical stress on the musculoskeletal system, there is a growing body of evidence, indicating that low-load resistance training also provides a potent training stimulus, when combined with blood flow restriction (BFR) (Kubota et al., 2008; Hackney et al., 2012; Schoenfeld, 2013; Lixandrão et al., 2018). The resistances used in BFR-training averages 30% of the one repetition maximum (1RM) which is well below the lower limits recommended for strength training (Schoenfeld, 2013). Nevertheless, muscle mass and strength gains induced by BFR-training, are comparable to that of high-intensity training regimen (Loenneke et al., 2012b). For use in microgravity, lower resistances during training would have the advantage of reducing the risk of strain-induced injuries for astronauts (Scheuring et al., 2009; Gabbett, 2016). Furthermore, it would have implications for the equipment required for training under microgravity conditions, as significantly lower resistances have to be provided by the training machines. This would comply with challenges of future space missions as vehicle resources, intra- and extravehicular physical constraints or access to earth-based monitoring (Loerch, 2015). Moreover, BFR has been shown to place a potent, gravitation-like stimulus on the cardiovascular system which may reduce the orthostatic intolerance upon the return to Earth (Iida et al., 2007; Nakajima et al., 2008).

This review article summarizes the possible advantages and disadvantages of BFR training in microgravity to counteract muscle atrophy, discusses the underlying mechanisms, and addresses the question of whether bones and tendons could also benefit from BFR training.

## Effects of Microgravity on the Musculoskeletal System

Prolonged mechanical unloading is well known to result in a significant de-adaptation of the musculoskeletal system (Lloyd et al., 2014). Muscle atrophy is thought to primarily result from a decreased protein synthesis based on a reduced activation of the IGF1-Akt-mTOR and the FAK-Akt-mTOR pathways (Gao et al., 2018). While the former seems to result from an unloading associated insulin resistance and an impaired insulin-like growth factor (IGF-1) signaling, the latter signaling pathway is directly affected by the elimination of mechanical stress, as the mechanosensitive focal adhesion kinase (FAK) is no longer activated (Graham et al., 2015). Bone loss is mainly driven by an altered differentiation of mesenchymal stem cells by an impaired integrin/mitogen protein kinase pathway due to mitogen-activated protein kinase (MAPK) (Yang et al., 2005). Beside integrins, osteocytes perceive mechanical stress via interstitial fluid, causing a biological cascade which results in WNt/β-catenin signaling that triggers bone remodeling (Rochefort and Benhamou, 2013). This and the dysfunction of osteoblasts, expressed due to reduced osteoblast proliferation and activity as well as a reduced cell differentiation lead to an impaired bone formation (Arfat et al., 2014). In comparison to muscle and bone, tendons have so far received little attention regarding adaptation to space flight. However, it is known that tendons adapt to the load that they have to withstand (Vanderby et al., 1990). With the lack of mechanical stress, as it appears in microgravity, proteoglycan and collagen synthesis get inhibited, leading to changes in structure (loss of diameter and density) and in chemical compositions (Johnson et al., 2005).

## In-Flight Exercise Protocols

In-flight exercise protocols are generally designed to minimize the loss in aerobic capacity, bone, muscle strength and endurance and to counteract neuromuscular dysfunction. The main goal thereby is to maintain in-flight and post-flight performance capabilities of the astronauts (Loehr et al., 2015). Crewmembers are commanded to adhere to their personal exercise protocols, including resistance (ARED) and cardiovascular exercise on a Treadmill or Veloergometer with Vibration Isolation and Stabilization System (TVIS, CEVIS). The training devices save personal data as well as physiological and training parameter, which allows the Mission Control Center (based on Earth) to adjust individual exercise schedules. Since the installation of the ARED in the International Space Station Expedition 18, high resistances can be applied during strength training on the ISS and the device allows about 29 different exercises. However, the ARED is very space-consuming and carries the potential risk of being temporarily unavailable due to technical faults (Hanson et al., 2014; Loehr et al., 2015), which motivates the search for and the exploration of smaller and technically simpler devices (Behringer et al., 2016). In addition, high training intensities are associated with an increased risk of injury to the musculoskeletal system (Gabbett, 2016), a fact to be taken seriously, as training-related injuries are the most common source of injury to astronauts on board the ISS (Scheuring et al., 2009). Therefore, the question arises whether BFR training can be a reasonable alternative or supplement for in-flight training sessions. In the following sections, the effects of primarily mechanical stimuli on the musculature are briefly presented and compared with those of more metabolically accentuated stimuli through BFR training.

## High Mechanical Tension as Training Stimulus for Muscles, Bones, and Tendons

High mechanical tension is well known as a potent stimulus to trigger muscle growth (Spurway and Wackerhage, 2006; Schoenfeld, 2010), bone mineral accrual (Bolam et al., 2013), and tendon stiffness (Brumitt and Cuddeford, 2015). In case of skeletal muscles, the mechanical forces are converted into intracellular anabolic signals by mechanosensors that are sensitive to the magnitude and the duration of the applied external force (Greenberg et al., 2016). Downstream processes are thought to be regulated by Akt/mTOR pathway (Latres et al., 2005), whereby mechanical tension stimulates mammalian target of rapamycin (mTOR) directly (Hornberger et al., 2006) or p70^S6K^ is phosphorylated (independent of mTOR) by phosphatidic acid (Lehman et al., 2007). Both pathways increase the protein synthesis of skeletal muscle cells. Furthermore, evidence suggests that mechanical tension activates the mechanosensitive FAK, which upregulates mTOR and thereby the protein synthesis (Chen et al., 1996; Bloch and Gonzalez-Serratos, 2003). However, the fact that high-intensity strength training is often accompanied by neuronal adjustments but only slight increases in muscle growth. Behm (1995) suggests that muscular tension alone cannot be responsible for muscle growth. Beside mechanical tension, stretch, cell swelling, systemic hormonal release, hypoxia, muscle damage, and ROS production are discussed as further reasons, activating anabolic signaling in skeletal muscle cells (Spurway and Wackerhage, 2006; Ozaki et al., 2015; de Freitas et al., 2017).

High mechanical forces placed on the musculoskeletal system result in bone matrix deformations inducing shear stress by bone fluid perturbations and cell membrane deformations through tethering elements of the glycocalyx (Bonewald and Johnson, 2008). Fluid flow, as well as intramedullary pressure are supposed to be influenced by mechanical loading, as well as vascular blood pressure, resulting in changing anabolic stimuli (Qin et al., 2003; Stevens et al., 2006). This mechanical stress is sensed by osteocytes (sensor cells) that transmit the signal to osteoblasts and osteoclasts (effector cells), ultimately stimulating bone formation on both, trabecular and cortical bone (Fujimura et al., 1997; Mi et al., 2005; Fritton and Weinbaum, 2009). Evidence is given, that biomarkers of bone formation like osteocalcin or bone-specific alkaline phosphatase (B-ALP) are increased after resistance training. Especially high training loads correlate with this response (Fujimura et al., 1997; Hu et al., 2011). However, since bone cells rapidly desensitize from mechanical stimuli intermittent loading regimens are necessary to allow for a resensitization of mechanoreceptors (Robling et al., 2002a,b; Saxon et al., 2005). Recent investigations expect the wingless-type (Wnt)/β-catenin canonical signaling pathway to be an important regulator in this process (Rochefort and Benhamou, 2013). While in osteoblasts, this pathway is crucial for synthesis, proliferation, and differentiation of the bone matrix, it enables osteocytes to transmit the sensed mechanical signals to cells on the bone surface.

Similar to the mechanisms in muscles and bones, mechanical tension in the tendon leads to the activation of mechanotransduction pathways, causing anabolic tissue responses (Arampatzis et al., 2009). Depending on the duration, frequency and intensity of the mechanical stimulus, the matrix protein synthesis, the expression and arrangement of collagen fibers as well as the expression of proteoglycans are adapted (Arampatzis et al., 2007). According to Arampatzis et al. (2007), the applied mechanical tension needs to exceed a certain threshold to induce adaptations of mechanical and morphological properties. This is supported by Kubo et al. (2006), who found that high- but not low-load isokinetic training of the knee extensors increased the stiffness of the vastus lateralis tendon–aponeurosis.

## Metabolic Stress as an Anabolic Signal for the Musculature

The mechanisms underlying the BFR-mediated muscle mass and strength gains still remain unclear. Since the mechanical load during this type of resistance training is low, it is assumed that the metabolic stress is primarily responsible for the induced adaptations. This is supported by the observation of Takada et al. (2012) who reported that hypertrophy and strength gains were correlated with the decrease of the intramuscular pH (hypertrophy: *r* = 0.80; strength gains: *r* = 0.65) and the accumulation of inorganic phosphate (hypertrophy: *r* = 0.88; strength gains: *r* = 0.60) during low-intensity (20% 1RM) BFR-training.

BFR-associated metabolic stress is a consequence of decreased oxygen supply caused by reduced blood flow (Kon et al., 2012), resulting in an impairment of the aerobic metabolism and premature fatigue of the aerobic slow-twitch fibers (Scott et al., 2014). Despite low external loads, the skeletal muscle is forced under these conditions to recruit fast-twitching muscle fibers to maintain force output, which further aggravates the accumulation of metabolites (Loenneke et al., 2011a). The accumulated metabolites are thought to provoke a reflex inhibition of alpha-motoneurons via type III and IV afferents resulting in a further increase of type II motor unit recruitment (Scott et al., 2014). Some authors see the recruitment of fast-twitch fibers as one of the central mechanisms by which BFR can trigger hypertrophy (Pope et al., 2013; Pearson and Hussain, 2015). Others believe that the acute release of anabolic hormones such as the human growth hormone (Abe et al., 2012; Pope et al., 2013; Park et al., 2015) or IGF-1 (Loenneke et al., 2011a; Scott et al., 2014; Park et al., 2015; Pearson and Hussain, 2015) contributes significantly to the BFR-mediated effects on muscle growth. The release of growth hormone appears to be associated with metabolic stress via the metaboreflex. This reflex is caused by locally accumulated metabolites activating metaboreceptors, which in turn activate the hypothalamic-pituitary axis via type III and IV afferents (Inagaki et al., 2011). Acute releases of catecholamines (e.g., norephrine response) have also been discussed as a factor for the exercise induced protein synthesis (Pope et al., 2013). However, several researchers have questioned the role of such acute exercise-induced hormone releases for muscle hypertrophy (Loenneke et al., 2012c; Pope et al., 2013).

The accumulation of osmotically active metabolites as lactate further leads to swelling of the muscle fibers as fluid shifts from the extra- to the intracellular space to equilibrate the osmotic gradient (Schoenfeld and Contreras, 2014). The resulting intracellular pressure is sensed by integrin-associated, cell-intrinsic volume sensors that activate mTOR and MAPK pathways, by which cell swelling is thought to trigger the muscular protein synthesis (Low et al., 1997; Abe et al., 2012; Pearson and Hussain, 2015). Kim et al. (2017) hypothesize that this muscle cell swelling induced pathway is one of the key mechanisms by which low-intensity BFR-training is able to induce anabolic effects (Loenneke et al., 2012a).

Another mechanism that could support BFR training induced muscle growth is the effect of reactive hyperemia on the vascular system. Two-fold increases in blood flow after BFR training over a period of more than 1 h have been reported (Gundermann et al., 2012). It is assumed that this long-lasting shear stimulus is responsible for the improved dilatory capacity of resistance vessels following BFR-Training (Hunt, 2014). In addition, BFR training increases microvascular filtration capacity as a sign of increased capillarization (Evans et al., 2010). Since adequate perfusion of the muscle fibers is crucial for muscle growth (Snijders et al., 2017), BFR-associated hyperemia with its effects on the vascular system could be an important factor supporting training-induced hypertrophy.

Furthermore, the ischemic conditions due to BFR lead to an upregulation of endothelial NOS, mRNA, and hypoxia inducible factor1α (HIF-1α), which influence autocrine factors (IGF-1) and satellite cell activation and thus, lead to increased protein synthesis (Pope et al., 2013; Pearson and Hussain, 2015). There are also some investigations that consider reactive oxygen species, increased glycogen storage or reduced myostatin to be influencing factors for muscle protein synthesis (Pope et al., 2013; Pearson and Hussain, 2015). However, there is no clear evidence for those factors. For example, it is well known that ROS production increases when blood supply returns (reperfusion) after sustained ischemia (Korthuis et al., 1985; Tsutsumi et al., 2007). Based on these observations, it could be assumed that the BFR-associated ischemia-reperfusion sequence exacerbates the hypoxic signaling cascade (dependent on HIF-1α). However, the data available so far often show no increase in ROS as a result of BFR training (Goldfarb et al., 2008; Rozales Ramis et al., 2017), so that the question of usefulness of antioxidant administration cannot yet be conclusively clarified.

## Effects of Low-Intensity BFR-Training on Bone Health

The majority of available literature on BFR training has dealt with its effects on skeletal muscle fibers, while only a few studies have investigated the effects on other tissues of the musculoskeletal system. However, some evidence is available that low-intensity BFR-training positively affects bone metabolism, formation and resorption (Bittar et al., 2018). Increased intramedullary pressure and interstitial fluid flow within the bone, caused by vascular occlusion, are hypothesized to be the main mechanisms affecting bone remodeling (Loenneke et al., 2012d). The effectiveness of BFR as a countermeasure for the bone loss was investigated by an increase of B-ALP, which is considered to display the activity of osteoblasts (Beekley et al., 2005). Further, bone resorption markers as C-terminal cross-linking telopeptide of type I collagen (CTX/NTX) has been reported to be decreased after BFR exercise (Bemben et al., 2007). Karabulut et al. (2011), who compared serum concentrations of bone markers in older man following high-intensity resistance training and low-intensity BFR-training, found B-ALP and B-ALP to CTX ratio improved after both training protocols. Although these results indicate that high mechanical loads are not necessary to prevent bone loss in microgravity, further research is needed to develop a better understanding of the BFR-training mediated effects.

## Effects of Low-Intensity BFR-Training on Tendons

There is some evidence that hypoxic conditions improve the proliferation of human tendon stem cells, when compared to normoxic conditions (Lee et al., 2012; Millar et al., 2012; Huang et al., 2013; Zhang and Wang, 2013; Jiang et al., 2014). Furthermore, hypoxia has been reported to be essential for the healing of the bone-tendon junction in which HIF-1α plays a key role (Zhao et al., 2011). Although, there are studies that have used BFR training in the rehabilitation of tendon injuries, the researchers’ main aim in those studies was to use BFR training to reduce the required training intensity to improve muscle strength (Yow et al., 2018). To the best of the authors’ knowledge, only few data are available regarding the effects of BFR training on the structure and function of tendons. In one study, Mohmara et al. (2014) investigated the effect of low-intensity (30% 1RM) leg-calf-raises either with or without BFR on the Achilles tendon thickness. The authors found no difference in tendon thickness between both conditions immediately and 24 h after the exercise protocol. However, this acute reaction does not provide any information about chronic tendon adaptations to BFR training programs. Kubo et al. (2006) reported that a 12-week resistance training (3 days/week) improved the stiffness of tendon-aponeurosis complex in the vastus lateralis only in the high-intensity (80% 1RM) but not in the low-intensity (20% 1RM) BFR group. Thus, according to the available literature it seems that high-mechanical forces cannot be dispensed with, if adaptations of the tendons are wanted. However, the data available on this topic are still very weak, so that further studies are required in order to be able to make reliable statements on this issue.

## Potential Risk Factors of BFR in Space

The risk of negative side effects of BFR-training has already been reviewed by others, whereby bruising under the cuffs (13.1%) due to the applied cuff pressure was most common (see, Loenneke et al., 2011b). In this regard, there is some disunity about the required cuff pressure and width for an optimal training response with as little vascular stress as possible. However, due to the potential side effects, there is broad agreement that individual cuff pressures should be preferred over fixed cuff pressures (Cook et al., 2007; Clark et al., 2011). Furthermore, there is some evidence that low cuff pressures (∼50% of the individual occlusion pressure) are sufficient to provoke the desired BFR-mediated effects on the musculoskeletal system and reduces the risk for negative side effects associated with higher pressures near arterial occlusion decrease (Loenneke et al., 2014a). Based on these arguments, it becomes clear that the measurement of the individual occlusion pressure of the astronauts is necessary to standardize the pressure of the cuff. Given the fact that blood pressure behaves differently under microgravity conditions (Norsk, 2014), pre-flight measurements are unsuitable for determining the individual occlusion pressure for training in space. Fortunately, BFR equipment is now available that automatically measures the individual closing pressure and adjusts the cuff pressure for the training accordingly. It is also conceivable that BFR training damages the muscles distal to the cuff. However, the small increase in muscle damage markers after BFR training speaks against this assumption (Loenneke et al., 2014b). One reason for the low level of damage is certainly the use of low resistance in this training method. Apparently, however, the induced ischemia is also not strong enough to have a direct or indirect (via. reperfusion injury) damaging effect on the muscle tissue (Thiebaud et al., 2013; Loenneke et al., 2014b). Reperfusion injury is caused by completely occluded blood flow to a limb, whereas the intensity and duration are pivotal role. In muscle tissue, irreversible damage can be seen after 4–6 h of occlusion (Blaisdell, 2002) and therefore the injury risk during BFR training is considered to be low. Nevertheless, there are also a few contradictory findings. Some authors reported that BFR increased the perceived muscle soreness as well as the sarcolemma permeability (Wernbom et al., 2012) and reduced the endothelial function (Renzi et al., 2010). In summary, however, the majority of the data indicate that the risk of muscle damage from BFR training is low.

Another common concern is the coagulation of blood and formation of thrombi by the BFR-induced disturbance of the laminar blood flow. Surprisingly, however, fibrinolytic activity has been reported to be increased after BFR training and the incidence of thrombosis to be lower compared to the general population (Nakajima et al., 2006). Other cardiovascular risk factors of BFR training are related to the decreased venous blood return to the heart. As a consequence, the heart rate and blood pressure increase to maintain cardiac output (Takano et al., 2005). This might be an important risk factor for people with an increased predisposition to cardiovascular disease. However, since astronauts are under strict medical supervision and are only allowed to fly into space if pre-flight medical examinations have been passed, this risk appears to be low for astronauts.

## Conclusion

Microgravity exposure has degenerative effects on the musculoskeletal system. Regarding further long duration flights like future Mars Expeditions, there is a need to tweak existing exercise protocols, to gain maximum training effects by using minimal equipment. Low load BFR-training allows for muscle mass and strength gains without the risk of injury associated with high resistances. Additionally, some evidence is available that bone mass and density can be increased by BFR exercise.

Despite the numerous positive findings on low-intensity strength training with BFR, it should be noted that these data were collected under normobaric and normoxic conditions. Therefore, future studies should clarify whether a hypobaric hypoxia (as may occur on board of future Mars Expeditions) or blood redistribution caused by weightlessness has an influence on BFR training results. It seems plausible that under these conditions lower cuff pressures are sufficient to trigger the effects of BFR training.

The low resistances required to achieve these goals also have an advantage regarding the equipment. Exercise devices would have to provide lower resistances, which facilitates their construction and preserves the vehicle capacity. Unfortunately, to date very little data are available whether low-intensity BFR-training is able to avoid the unloading associated deterioration of tendons. Nevertheless, the results of the review clearly show that BFR training is a useful supplement to training in microgravity.

Future studies are needed to investigate whether the blood redistribution caused by weightlessness has an influence on BFR training results. Therefore, long-term, 6° head down tilt bed rest studies investigating BFR-training should be sought to evaluate physiological adaptations of the musculoskeletal system.

## Author Contributions

MB and CW did the research for this topic, the framework and structure of this article, and discussed the possible effects.

## Conflict of Interest Statement

The authors declare that the research was conducted in the absence of any commercial or financial relationships that could be construed as a potential conflict of interest.
