# Corrigendum: Application of Blood Flow Restriction to Optimize Exercise Countermeasures for Human Space Flight

**DOI:** 10.3389/fphys.2019.00276

**Published:** 2019-03-26

**Authors:** Michael Behringer, Christina Willberg

**Affiliations:** Institute of Sports Sciences, Goethe University Frankfurt, Frankfurt, Germany

**Keywords:** human space flight, exercise countermeasure, adaptations to microgravity, BFR training, space adaptations

In the original article, there was an error. The International Space Station Expedition was incorrectly referred to as “the Skylab Expedition 18.”

A correction has been made to the **In-Flight Protocols** section:

“In-flight exercise protocols are generally designed to minimize the loss in aerobic capacity, bone, muscle strength and endurance and to counteract neuromuscular dysfunction. The main goal thereby is to maintain in-flight and post-flight performance capabilities of the astronauts (Loehr et al., 2015). Crewmembers are commanded to adhere to their personal exercise protocols, including resistance (ARED) and cardiovascular exercise on a Treadmill or Veloergometer with Vibration Isolation and Stabilization System (TVIS, CEVIS). The training devices save personal data as well as physiological and training parameter, which allows the Mission Control Center (based on Earth) to adjust individual exercise schedules. Since the installation of the ARED in the International Space Station Expedition 18, high resistances can be applied during strength training on the ISS and the device allows about 29 different exercises. However, the ARED is very space-consuming and carries the potential risk of being temporarily unavailable due to technical faults (Hanson et al., 2014; Loehr et al., 2015), which motivates the search for and the exploration of smaller and technically simpler devices (Behringer et al., 2016). In addition, high training intensities are associated with an increased risk of injury to the musculoskeletal system (Gabbett, 2016), a fact to be taken seriously, as training-related injuries are the most common source of injury to astronauts on board the ISS (Scheuring et al., 2009). Therefore, the question arises whether BFR training can be a reasonable alternative or supplement for in-flight training sessions. In the following sections, the effects of primarily mechanical stimuli on the musculature are briefly presented and compared with those of more metabolically accentuated stimuli through BFR training.”

The authors apologize for this error and state that this does not change the scientific conclusions of the article in any way. The original article has been updated.
